# Multi-Scale Expressions of One Optimal State Regulated by Dopamine in the Prefrontal Cortex

**DOI:** 10.3389/fphys.2019.00113

**Published:** 2019-02-28

**Authors:** Guyue Hu, Xuhui Huang, Tianzi Jiang, Shan Yu

**Affiliations:** ^1^Brainnetome Center, Institute of Automation, Chinese Academy of Sciences, Beijing, China; ^2^National Laboratory of Pattern Recognition, Institute of Automation, Chinese Academy of Sciences, Beijing, China; ^3^Research Center for Brain-inspired Intelligence, Institute of Automation, Chinese Academy of Sciences, Beijing, China; ^4^CAS Center for Excellence in Brain Science and Intelligence Technology, Chinese Academy of Sciences, Beijing, China; ^5^University of Chinese Academy of Sciences, Beijing, China

**Keywords:** optimal states, working memory, criticality, E/I balance, dopamine, the PFC

## Abstract

The prefrontal cortex (PFC), which plays key roles in many higher cognitive processes, is a hierarchical system consisting of multi-scale organizations. Optimizing the working state at each scale is essential for PFC's information processing. Typical optimal working states at different scales have been separately reported, including the dopamine-mediated inverted-U profile of the working memory (WM) at the system level, critical dynamics at the network level, and detailed balance of excitatory and inhibitory currents (E/I balance) at the cellular level. However, it remains unclear whether these states are scale-specific expressions of the same optimal state and, if so, what is the underlying mechanism for its regulation traversing across scales. Here, by studying a neural network model, we show that the optimal performance of WM co-occurs with the critical dynamics at the network level and the E/I balance at the level of individual neurons, suggesting the existence of a unified, multi-scale optimal state for the PFC. Importantly, such a state could be modulated by dopamine at the synaptic level through a series of U or inverted-U profiles. These results suggest that seemingly different optimal states for specific scales are multi-scale expressions of one condition regulated by dopamine. Our work suggests a cross-scale perspective to understand the PFC function and its modulation.

## Introduction

The brain is consisting of structures at different scales that are hierarchically organized, ranging from synapses and cells all the way to networks of brain areas (Park and Friston, 2013; Betzel and Bassett, 2017). Incorporating regularities for different levels to give a coherent, cross-scale account for brain functions is a significant challenge for systems neuroscience. The prefrontal cortex (PFC), which is involved in many higher cognitive processes, such as working memory (WM), planning, and multi-tasking (Yang and Raine, 2009; Diamond, 2013), has been intensively studied at different scales, revealing diverse scale-specific optimal states that can benefit the information processing occurring at corresponding scales. Firstly, at the system level, WM, which refers to the ability to temporarily hold and manipulate information in the brain (Baddeley, 1992, 2012), is strongly modulated by dopamine (DA) according to a well-established “inverted-U” profile. That is, too strong or too weak of dopamine D1 activation is detrimental for WM, with optimal performance achieved at an intermediate level (Zahrt et al., 1997; Vijayraghavan et al., 2007). Deficits in this modulation can lead to severe impairment in WM, which is a key symptom in various brain disorders (Austin et al., 2001; Steele et al., 2007), such as schizophrenia (Lett et al., 2014). Secondly, at the network level, it has been discovered that the state of PFC networks *in vitro* could be affected by DA. That is, intermediate dopamine D1 receptor activation led to a so-called critical state (Stewart and Plenz, 2006), which has been suggested as the optimal state for neuronal information processing (Beggs and Plenz, 2003; Kinouchi and Copelli, 2006; Levina et al., 2007; Millman et al., 2010; Yu et al., 2017). Thirdly, at the cellular level, the balance between the excitation and inhibition, reflected by the close tracking of the inhibitory inputs to the excitatory ones for individual neurons (Okun and Lampl, 2008), has been suggested as an important factor that modulates the overall working state of the network (Vogels et al., 2011). Although diverse biological and computational approaches (Cools and D'Esposito, 2011; Barak and Tsodyks, 2014) have been used to study the working state regulation in the PFC, it remains unclear whether the optimal states manifested at individual scales mentioned above are just different expressions of the same unified, cross-scale optimal state at the PFC.

In addition, if a unified optimal state indeed exists, what could be the underlying mechanism modulating it at all scales simultaneously? Anatomical studies indicated that the PFC contains many DA receptors (Goldman-Rakic, 1995) and receives diffuse projections from midbrain dopaminergic neurons (Robbins, 2000). Thus, a potential candidate for the cross-scale modulation of the optimal state is the dopamine modulation. Previous studies have shown that different degrees of dopamine D1 receptor activation act differentially on glutamatergic synapses between the excitatory and inhibitory neurons. Specifically, with low doses of DA, the inputs to both excitatory and inhibitory neurons are unaffected; with moderate doses of DA, the enhancement of glutamatergic input to excitatory neurons is more pronounced; and with high doses of DA, the inputs to both excitatory and inhibitory neurons are strongly enhanced (Muly et al., 1998; Gao et al., 2001). However, how such a mechanism could give rise to the modulation across different scales in order to adjust the working state of the PFC remains unclear.

Here we address these two issues by studying a network model. We found that the optimal performance of WM at the system level co-occurs with critical neuronal dynamics at the network level and the most balanced excitation and inhibition at the cellular level. Importantly, such a unified optimal state is obtained through an intermediate level of dopamine D1 activation at the synaptic level. These results suggest that empirically observed, seemingly different optimal states at individual scales are different expressions of one condition regulated by dopamine. These results shed new light on the multi-scale state optimization for information processing in the PFC.

## Results

### The State Transition of Neuronal Dynamics in the Network

**Figure 1:**
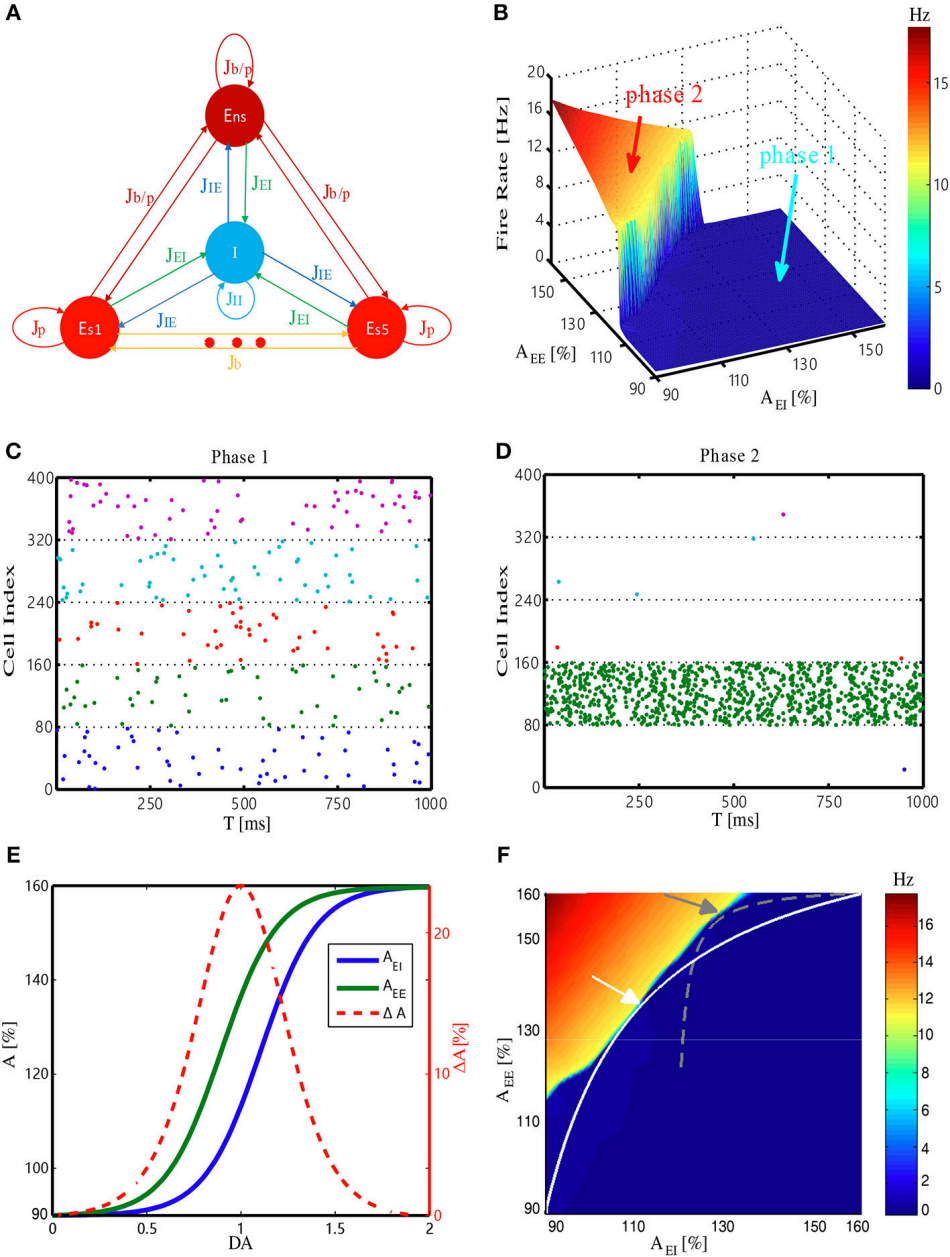
Structure and dynamic behavior of the WM model, and its DA modulation. **(A)** Network architecture. E_S1_, …, E_S5_, E_ns_ and I denote five selective excitatory populations, one non-selective excitatory population, and one inhibitory population, respectively. *J*_*ij*_ (where *i, j* is I or E) denotes the synaptic connection from *i* population to *j* population. The E–E synapse can be a potentiated value (*J*_*p*_), baseline value (*J*_*b*_), or potentiated value with a certain probability (*J*_*b*/*p*_). *J*_*EI*_ and *J*_*EE*_ (*J*_*b*_*, J*_*p*_*, J*_*b*/*p*_) are modulated by DA (see Materials and Methods). **(B)** Firing rate of the most active Es population at rest (i.e., without external stimulus) changes with synaptic strength among excitatory neurons (*J*_*EE*_) and from the excitatory to inhibitory neurons (*J*_*EI*_). *A*_*EE*_ and *A*_*EI*_ are the scaling factors for *J*_*EE*_ and *J*_*EI*_, respectively. **(C,D)** Spiking patterns corresponding to the two different phases (*phase1* and *phase2*) in **(B)**. Different colors represent five Es populations (only 10% neurons are shown), with each dot denoting a spike. **(E)** Scaling factors *A*_*EE*_ and *A*_*EI*_ change as a function of dopamine D1 activation level. The strength difference (Δ*A* = *A*_*EE*_−*A*_*EI*_) reaches its maximum at DA = 1.0. **(F)** Trajectories represent how the corresponding system state changes with DA. The system represented by the white trajectory is analyzed in the main text. Similar results can also be obtained through the gray trajectory (with a different synaptic strength range), demonstrating the robustness of the results. Arrows mark the intermediate level of DA = 1.0.

Our network model is adapted from a biologically plausible WM model (Mongillo et al., 2008). In this model, the external input for the network first activates one of the excitatory-selective neuronal populations (Es, cf. Figure 1A), whose activities form the internal representation of the input. These activities trigger short-term synaptic facilitation, resulting in the strengthening of the synaptic connections within this population. Consequently, a strongly interconnected neuronal group is temporarily formed. Through recurrent excitation, this group can maintain its activity as the internal representation of the recent input, even after the input is removed, thereby forming WM. To investigate how network's WM performance can be modulated, we examined its behavior within a 2-D parameter space (the EE–EI plane). The two dimensions represent synaptic strength among excitatory neurons (*J*_EE_) and strength of synapses from excitatory to inhibitory neurons (*J*_EI_), respectively. Driven by weak background noise, the average firing rate of the neuronal populations changed as a function of *J*_*EE*_ and *J*_*EI*_ (Figure 1B). In this EE-EI plane, we found phase transition from a low (*phase1*) to high activity regime (*phase2*). In *phase1*, the neuronal activities were very sparse, with weak responses evoked by background noise (Figure 1C), whereas in *phase2*, high-frequency reverberating activities within one population were maintained without external inputs (Figure 1D). Note that the active population in this case was stochastically chosen by the dynamics. This population activates the inhibitory group (I, cf. Figure 1A), resulting in the suppression of activities of other populations. Network behavior analysis within the EE-EI plane provided a clear view of how WM can be achieved. That is, in normal condition, the network resides in *phase1* at rest (i.e., without external input). When the external input triggers activities leading to short-term increases in *J*_EE_, the network state moves along a trajectory parallel to the *J*_EE_ axis and toward the phase transition border. If the input is sufficiently strong to push the system across the transition border into *phase2*, the reverberating activities are self-maintained and WM is formed. Contrastingly, if the network resides in *phase2* at rest, the maintained reverberating activities have no corresponding sensory event (“imaginary memory”), which is reminiscent of hallucination in brains disorders, e.g., schizophrenia (Horga et al., 2014; Llorca et al., 2016).

### Dopamine Modulation at the Synaptic Level in the Model

We next study how to model the dopamine modulation at the synaptic level and introduce it into the model. Previous studies have indicated that activation of the dopamine D1 receptor can have different effects on the excitatory inputs between excitatory to excitatory and excitatory to inhibitory synapses. Specifically, glutamatergic input of excitatory neurons may increase at low D1 activation and, such a strengthening effect saturates relatively early; however, glutamatergic input of inhibitory neurons is less sensitive to D1 activation, resulting in “delayed” onset and saturation of strengthening effects (Muly et al., 1998; Dash et al., 2007). Therefore, in our model, the effects of increasing the dopamine D1 activation level were simulated by changing the strengths of *J*_EE_ and *J*_EI_ through multiplying corresponding scaling factors *A*_*EE*_ and *A*_*EI*_, according to the functions shown in Figure 1E. For each level of D1 activation, we deduced the corresponding values of *J*_EE_ (*A*_*EE*_) and *J*_EI_ (*A*_*EI*_), providing coordinates to pinpoint the network state in the EE-EI plane. Eventually, a continuous trajectory representing how the network state changed was obtained (Figure 1F, white curve). With the increases in D1 activation level, the network initially approached and then deviated from the transition border, with the shortest distance achieved by an intermediate D1 activation level. This analysis was not sensitive to specific positions at which the trajectory met the phase transition border (determined by different strength range, see Materials and Methods), as similar results can be obtained with different trajectories (Figure 1F, gray curve).

### WM Performance at the System Level Regulated by Dopamine

**Figure 2:**
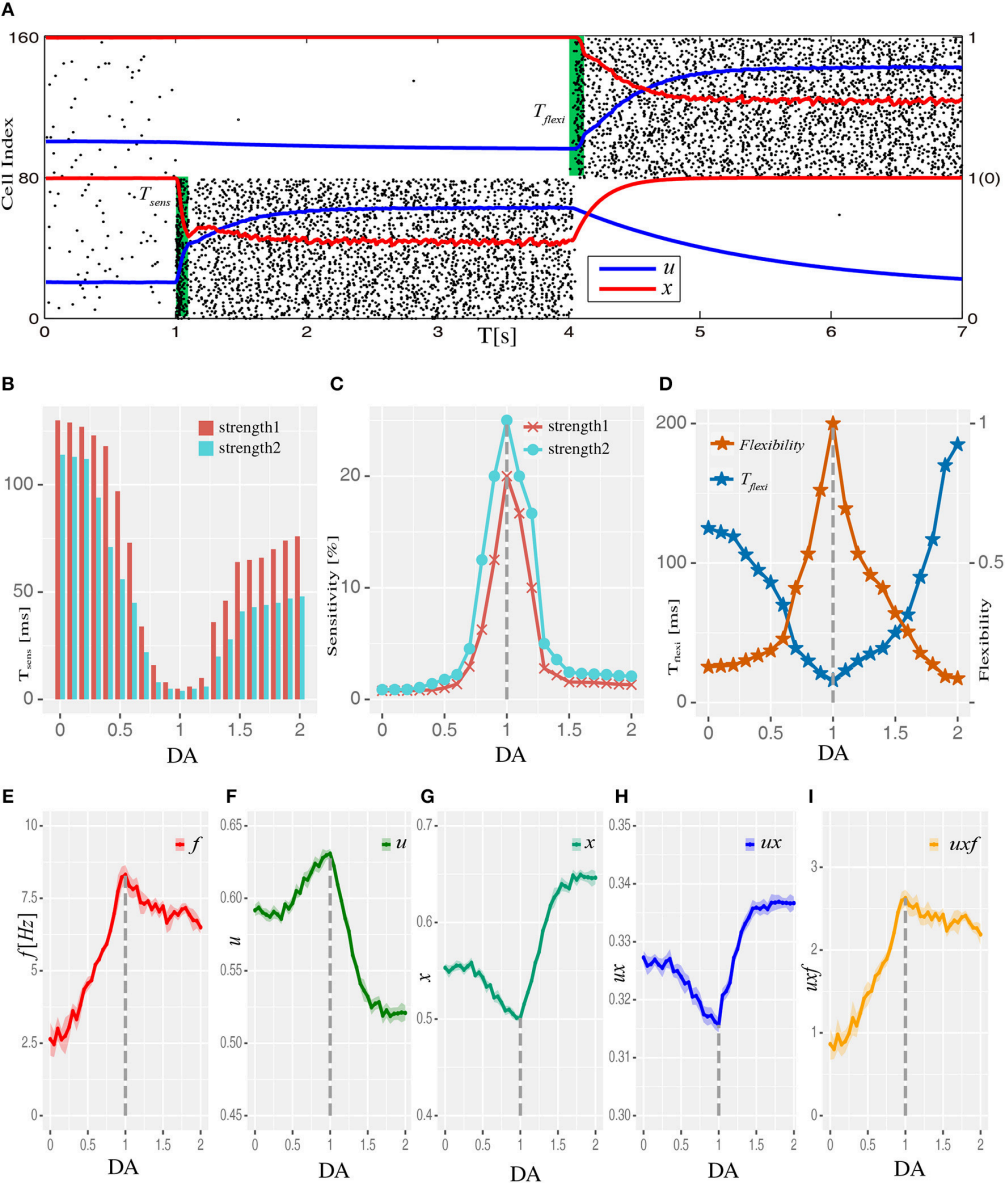
Dopamine modulation and the optimal working memory performance. **(A)** One item is *loaded* into memory from the resting state and *updated* to another item by applying item-specific stimuli (green shading) with a durations of *T*_*sens*_ and *T*_*flexi*_, respectively. Black dots are neural spikes (only 10% neurons are shown). *u* and *x* donates average utilization parameter and available resource in corresponding populations, respectively. **(B)** The shortest stimulation time (*T*_*sens*_) needed for the model to load the stimulus into WM from resting state at each D1 activation level. Results for two stimulus strengths (strength1 < strength2) are shown. **(C)** Sensitivity of WM, defined as the reciprocal of *T*_*sens*_, exhibits an inverted-U profile with dopamine D1 modulation. **(D)** The shortest time (*T*_*flexi*_) needed to update a memory item and corresponding flexibility were also shaped by the U and inverted-U profiles, respectively. **(E–I)** Internal parameters that determine the system's dynamics change as a function of D1 activation, including firing rate *f* (average firing rate), *u* (utilization parameter), *x* (available resources), *ux* (transmitters used per spike), and *uxf* (total transmitters used) of the population receiving memory stimuli (strength1, and in total 200 ms long), which was E_s1_ for all analyses presented in this paper. Data are represented as mean ± S.D. across all the neurons in the population.

We then examined how WM performance at the system level changed along the regulation trajectory of dopamine D1 activation mentioned above. As shown in Figure 2A, a memory item could be *loaded* into WM when the network was in the resting state. Importantly, the content of WM could also be *updated* if another item need to be memorized. At the “behavioral” level, these two measures were applied to evaluate the performance of WM, including the sensitivity of loading items and the flexibility of updating memory. Specifically, at each level of D1 activation, stimuli with a fixed strength were applied to one item-selective excitatory population (Es) with different durations. As shown in Figure 2B, we found that regardless of the input strength, the shortest time (*T*_*sens*_) needed for successfully *loading* an item into WM exhibited a U-shaped profile. As a result, the *sensitivity* of WM, defined as the reciprocal of *T*_*sens*_, had an inverted-U profile, with the maximal sensitivity achieved with the intermediate D1 activation (Figure 2C). Similarly, when we defined the shortest time needed to *update* a memory item as *T*_*flexi*_ (normalized by the smallest *T*_*flexi*_ obtained in the whole DA modulation curve), and used its reciprocal as the measure of the *flexibility* of WM, we found that the flexibility of WM also exhibited an inverted-U profile (Figure 2D).

Next we studied the network mechanisms underlying these WM behaviors. As WM is formed by the system jumping from *phase1* to *phase2* in response to an external input, the closer the original state is to the transition border, the easier it is for the system to go through the transition. Closer examination of the system's behavior revealed that intermediate D1 activation was associated with the highest firing rates *f* (Figure 2E) and maximal neural transmitter utilization parameter *u* (Figure 2F). Accordingly, in such a state, the total amount of available transmitter in presynaptic neurons, *x*, was lowest (Figure 2G). Interestingly, as the increase in *u* was less pronounced compared with the decrease in *x*, the amount of neural transmitter released per spike (*ux*) reached the minimum (Figure 2H), reflecting a more efficient use of transmitter to produce individual spikes in the network. However, as the increase in firing rates was more pronounced under an intermediate D1 activation, the overall use of neural transmitter in the entire network (*uxf*) was maximized in such a state (Figure 2I). This observation indicates the price the system needs to pay for increased sensitivity and flexibility of WM—the accelerated pace of consuming neurotransmitter and, consequently, more energy used to refill the reservoir. Note that the U or inverted-U profiles in Figure 2 are non-symmetrical. Although the difference of connecting strengths (Δ*A*) could be the same for lower and higher dopamine concentrations, the absolute connecting strengths (*A*_*EE*_, *A*_*EI*_) with higher D1 activations were larger than those with lower D1 activations (Figures 1E,F), which led to different network dynamics.

### Critical State at the Network Level Regulated by Dopamine

**Figure 3:**
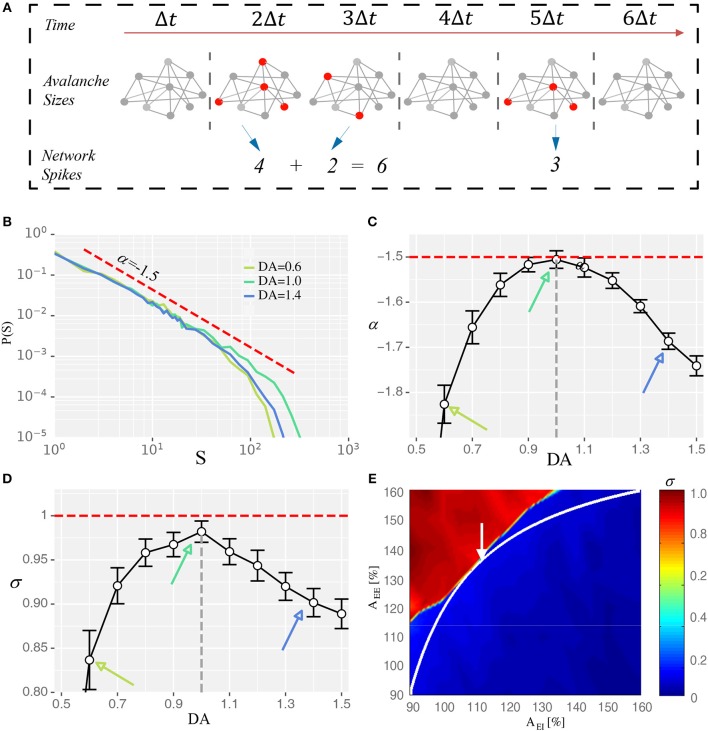
Intermediate D1 activation corresponds to a state close to criticality. **(A)** Schematic diagram showing the identification of neuronal avalanches. Solid circles represent individual neurons, with red donating active state and gray donating quiescence. Neurons that fire in the same time bin or consecutive bins of length Δ*t* form an activity cascade, i.e., an avalanche. Avalanche size, *s*, is defined as the number of active neurons in the cascade. Two avalanches (*s* = 6 and 3) are shown. **(B)** Avalanche size distribution obtained with three different levels of D1 activation, with the intermediate level (DA = 1.0) associated with distribution closest to a power-law exponent of −1.5 (red dashed line). **(C,D)** The exponent (α) and the branching parameter (σ) change as a function of the D1 activation level. Red dotted lines represent α = −1.5 and σ = 1 in **(C,D)**, respectively. States corresponding to DA = 0.6, 1.0, and 1.4 are marked by arrows with the same color as in **(B)**. α and σ estimated with different D1 activation levels are statistically significant (one-way ANOVA, *p* < 0.05; *post-hoc* test among DA = 0.6, 1.0, and 1.4, *p* < 0.05; Data are represented as mean ± S.D. across fifteen trials at each DA level). **(E)** Branching parameter (σ) exhibits a phase transition similar to the firing rate shown in Figure 1F.

To bridge the state modulation regularity of WM performance at the system level with the corresponding regulation processes and characteristics at the network level, we examined the relationship between the maximal WM sensitivity/flexibility and features of network's dynamics. Specifically, we analyzed two indicators of so-called critical dynamics: avalanche size distribution and branching parameter. Avalanches are activity cascades within the system and avalanche size is how many neurons are involved in the corresponding cascade (Figure 3A). A hallmark of the critical state is that avalanche size distribution exhibits a power-law with the exponent close to −1.5 (Beggs and Plenz, 2003). Consistently, we found the network dynamics exhibited a power-law distribution with exponent closest to −1.5 under the intermediate D1 activation (Figures 3B,C). Another indicator of critical dynamics is the branching parameter, which is defined as how many neurons, on average, can be activated by one active neuron. It measures how quickly the activities in a recurrent network are amplified or attenuated. Stable activity propagation of the critical state is associated with a branching parameter close to 1 (Beggs and Plenz, 2003; Shew and Plenz, 2013). Here, we found that the branching parameter estimated from the network dynamics was closest to 1 under the intermediate D1 activation (Figure 3D). Importantly, with too strong or too weak D1 activation, the system deviated from the critical state in the same direction. Specifically, large avalanches were formed less frequently and the branching parameter was <1, indicating a subcritical state in which the propagation of activities was over-attenuated. Such a phenomenon is in line with the previous finding that high or low D1 activation resulted in a subcritical state in brain slices (Stewart and Plenz, 2006), reflected by the deeper slopes of avalanche size distributions and branching parameters being smaller than one. Further, to provide an overview of the network state regarding the distance from criticality (Figure 3E), we plotted the branching parameter for all possible states within the EE-EI plane. We found a phase transition in the branching parameter corresponding to the transition in terms of network activity level, and the self-sustained activities in the top left part was associated with a branching parameter close to 1. The trajectory of DA modulation (Figure 3E) provides a direct assessment of how the distance from the critical state was modulated by different levels of D1 activation. Compared with Figure 2, it is clear that the critical state jointly emerged with the optimal WM performance when the intermediate degree of dopamine D1 activation (DA = 1.0) was set at the synaptic level.

### Criticality at the Network Level Maintained by E/I Balance at the Cellular Level

**Figure 4:**
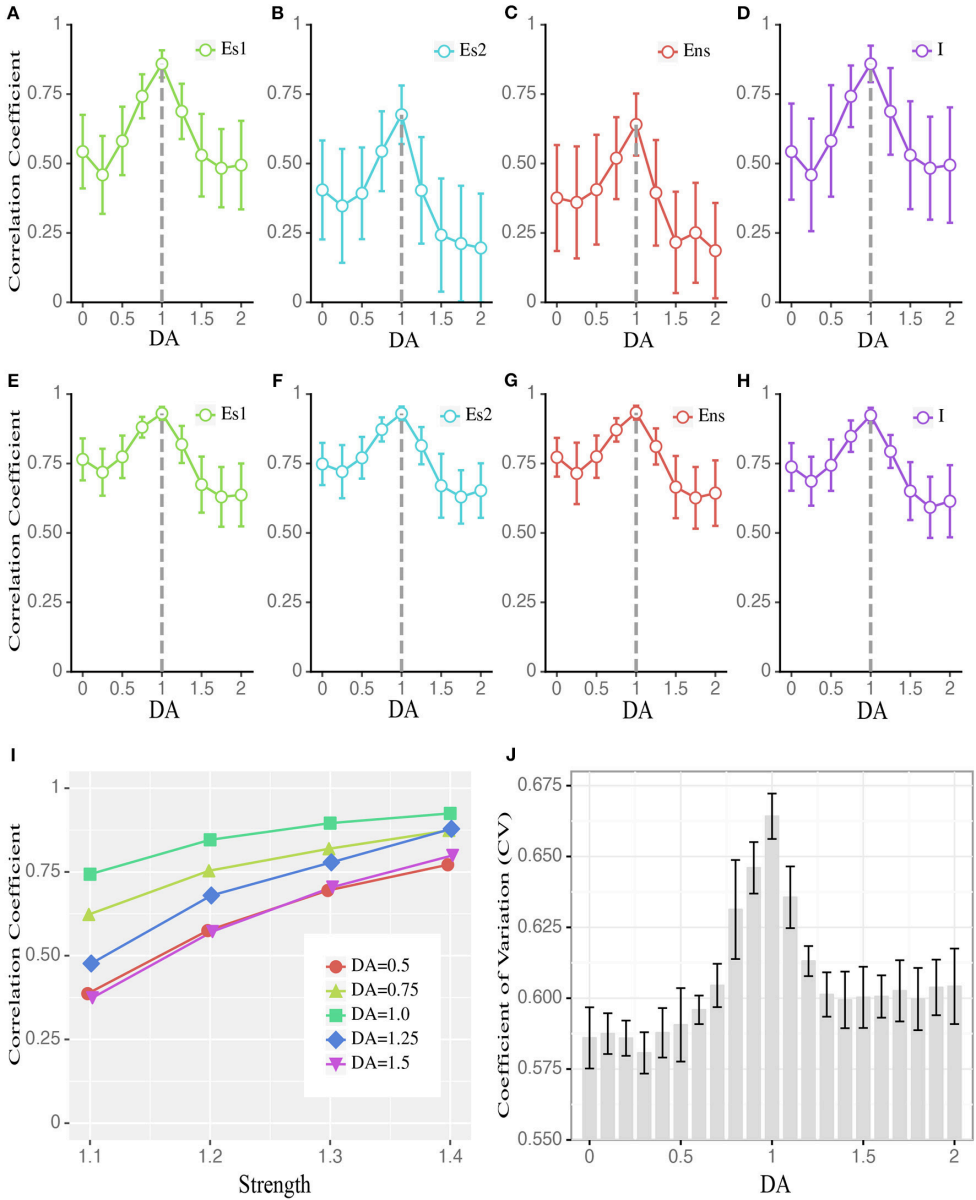
Dopamine modulates the balance between excitatory and inhibitory currents to individual neurons. **(A–D)** The average correlation coefficient of excitatory and inhibitory currents received by individual neurons in different populations during stimulus presentation, including stimulus-targeted selective population (E_s1_), Es population without stimulation (results for E_s2_ are shown as an example), non-selective population (E_ns_), and inhibitory population (I). Data are means ± SD across the corresponding population. **(E–H)** Similar to **(A–D)** but the correlation coefficient is computed based on only recurrent currents, excluding external inputs and leaky currents. **(I)** The correlation coefficient of E_s1_ (to save space, E_s2_, E_ns_, and I are omitted.) in **(A)** increases with stronger stimulation strengths. DA = 1 is associated with the largest correlation across all strengths. For visual clarity, only the means of the correlation coefficients across the corresponding population are shown. **(J)** CV (coefficient of variation) of the inter-spike interval changes as a function of DA modulation, with DA = 1 corresponding to the highest CV (Data are represented as mean ± S.D. across fifteen trials at each DA level). The group differences between low, high, and intermediate DA in **(A–J)** are statistically significant (one-way ANOVA with multiple comparison tests under Tukey's criterion, *p* < 0.05).

We next addressed how critical dynamics at the network level could be maintained with the intermediate degree of D1 activation. Specifically, given the short-term synaptic facilitation mechanisms built in the network, and that the intermediate D1 activation leads to the most active network state, what mechanism can prevent the system from a runaway excitation, i.e., being supercritical, under such a condition? One possible mechanism is the balance between the excitation and inhibition, i.e., E/I balance, at the cellular level. Reflected by the close tracking of the inhibitory inputs to the excitatory ones, E/I balance is well-documented (Okun and Lampl, 2008) and is suggested as an important factor modulating the overall network state (Vogels et al., 2011). This balance is essential to maintain the states of neuronal networks, demonstrated by both experimental (Shew et al., 2009) and modeling studies (Lombardi et al., 2012; Poil et al., 2012). Under the intermediate D1 activation, the correlation between the total inhibitory and excitatory currents in the network was maximized (Figure 4), reflecting a higher level of E/I balance. This effect was global to the entire network, as it was manifested in all neuronal populations (Figures 4A–D). Furthermore, the correlation between the E and I currents was higher when we only analyzed the recurrent inputs (i.e., without external inputs, background inputs, and membrane leakages; see Methods) (Figures 4E–H), suggesting that recurrent dynamics dominated the E/I balance. To further verify this, we examined how the E/I currents correlation changed as a function of recurrent activity levels induced by external inputs with different strengths. As shown in Figure 4I, this correlation increased monotonically as a function of the strength of external inputs, with the intermediate D1 activation associated with the highest correlation, reconfirming the casual role of recurrent activities in determining the E/I balance. Besides the highly correlated E and I currents, a large coefficient of variation (CV) of neuronal activities is also an important indicator of the E/I balance (van Vreeswijk and Sompolinsky, 1996). Consistently, we found that the CV of network activities was maximized with the intermediate D1 activation (Figure 4J). These results imply that the network state under the intermediate D1 activation is associated with the highest level of E/I balance, which allows the inhibition to track the excitatory drive within the network and to avoid the supercritical state.

### Multi-Scale Expressions of One Optimal State Regulated by Dopamine

**Figure 5:**
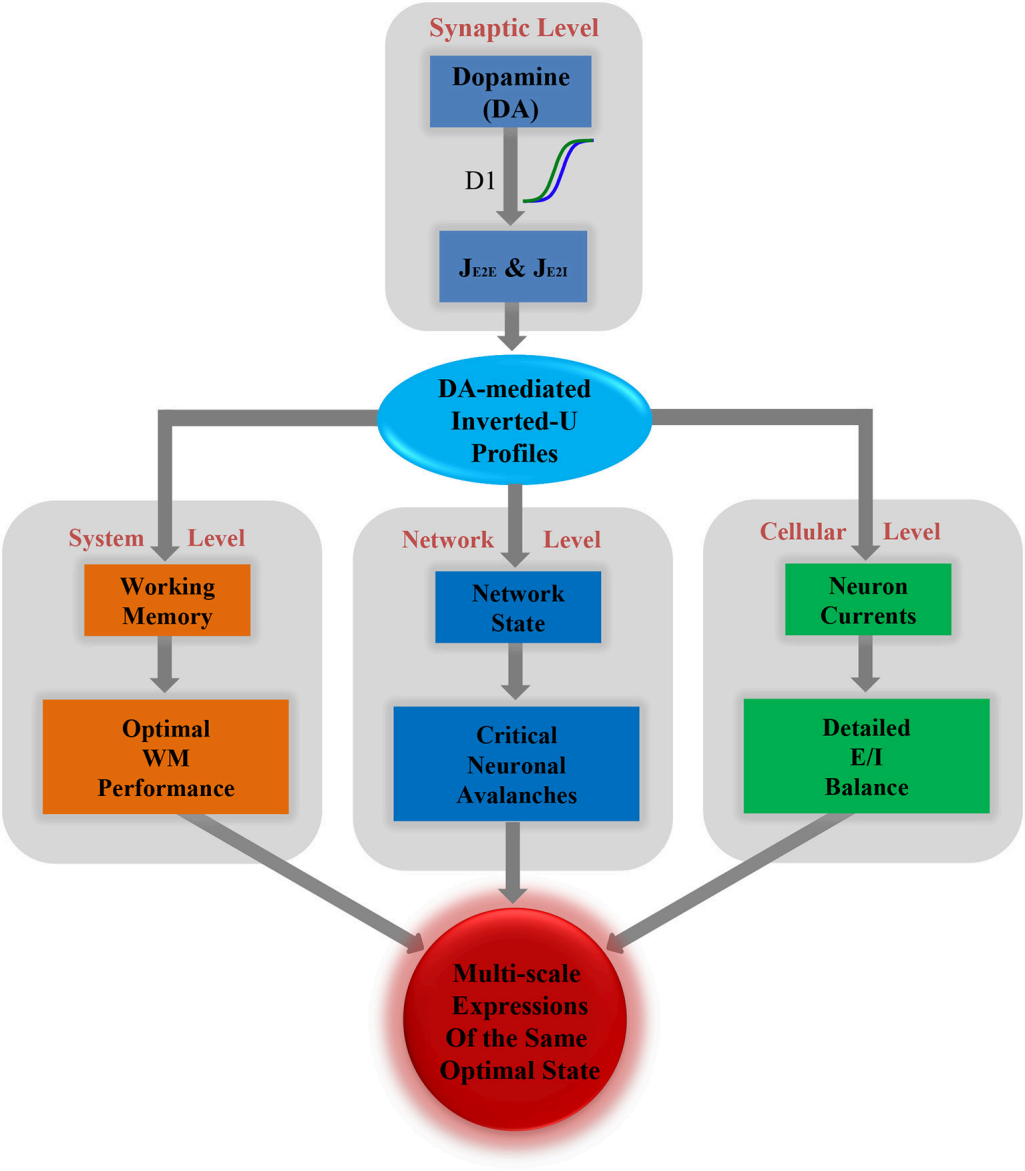
Multi-scale expressions of the same optimal state regulated by dopamine, including detailed E/I balance at the cellular level, critical dynamics at the network level, and optimal WM performance at the system level.

Finally, based on all the results described above, we propose a framework incorporating the optimal states from the perspectives of WM, network dynamics and the E/I balance, as shown in Figure 5. At the synaptic level, different levels of dopamine D1 activation have different enhanced efficacy to the glutamatergic input of the excitatory and inhibitory neurons, i.e., J_EE_ and J_EI_. This changes the relative strength of the excitation and inhibition in the system. As a result, the measures of WM, critical dynamics and the E/I balance at different scales are all shaped by a series of U or inverted-U profiles. The extrema of these U or inverted-U profiles suggest optimal working states for corresponding scales, which simultaneously obtained by the intermediate level of D1 activation. According to such a framework, the optimal states in the PFC, including the best WM performance at the system level, critical neuronal dynamics at the network level, and detailed E/I balance at the cellular level are multi-scale expressions of one state modulated by dopamine.

## Discussion

When interpreting the results, several limitations need to be clarified. Firstly, we did not consider the lags between the excitatory and inhibitory currents in calculating their correlations. From previous current-clamp recordings in single neurons, the inhibitory currents lag about a few milliseconds behind the excitatory currents (Okun and Lampl, 2008). Such values are smaller than the sampling interval of currents in our simulation. Therefore, neglecting the lags in calculating the correlation between excitatory and inhibitory currents would not have a strong effect on the results. Secondly, in our simulation, we chose a specific range of A_EE_ and A_EI_, however, the main results obtained can be well-replicated by other choices of the range of A_EE_ and A_EI_, e.g., the results (cf., Supplementary Figure S1) obtained from the gray modulation curve in Figure 1F, suggesting that our results are generalizable for different dopamine baseline and modulation range. Thirdly, many different computational models of WM have been proposed (Wang, 2002; Goldman et al., 2003; Machens, 2005; Barak and Tsodyks, 2014). Here we built our system by adapting a biophysically realistic model of WM (Mongillo et al., 2008), which is agreed well with various empirically observed electrophysiological properties (Rainer and Miller, 2002; Shafi et al., 2007). It awaits future studies to investigate if the same results can be obtained in other WM models. Fourthly, in the present model, the neural noise was modeled as stable Gaussian white noises, as in many other computational models of WM (Brunel and Wang, 2001; Mongillo et al., 2008). Recently, it was reported that the variance of neural noise is related to environmental factors, such as body temperature (Dvir et al., 2018), which has not been considered here. Our results demonstrated that it was the recurrent currents rather than the background noise primarily determined the detailed E/I balance for individual neurons (Figures 4A–I). Thus, as long as the noise variance level is within a normal range (i.e., the noise currents do not suppress the recurrent currents), the optimal state would not change.

In our results, the optimal working memory performance is regulated by dopamine by a typical U or inverted-U profiles, which are in line with many previous empirical (Dash et al., 2007; Vijayraghavan et al., 2007; Kroener et al., 2009; Van Snellenberg et al., 2016) and computational (Brunel and Wang, 2001; Dash et al., 2007; Lew and Tseng, 2014) studies . On top of that, our results provide a possible link between the system level characteristics and underlying synaptic mechanisms. We show that not only the measures at the system level like sensitivity and flexibility but also the measures at the synaptic level, such as available resources (*x*), are shaped by dopamine-mediated U or inverted-U profiles. These results indicate that the modulation of WM performance at the system level is an aggregated effect of modulations occurring at the finer scales of neural networks.

A neural network operating close to a critical state has various functional advantages in terms of information encoding, storage, transmission, and processing (Kinouchi and Copelli, 2006; Beggs, 2008; Kello, 2013). Along with the empirical evidence that biological neural networks were indeed exhibited typical behavioral hallmarks of criticality in their dynamics, it has been long expected that critical neural networks can play an essential role in various brain functions, and the deviation from such a state may lead to functional deficits as seen in many brain disorders. However, so far there is no mechanism to directly link the critical neural network and well-characterized brain functions. Our results demonstrate that the optimal performance of working memory at the system level was achieved when the neural network was operating most close to a critical state at the network level, and deviation from the critical state would impair WM performance. Because the basal dopamine levels are variable across individuals (Mattay et al., 2000; Gibbs and D'Esposito, 2005; Cools and D'Esposito, 2011), the DA-induced inverted-U profile of WM performance is not a robust biomarker for diagnosing working memory deficits. Unlike the optimal concentration suggested by the DA-induced inverted-U profile of WM performance, which varies greatly across individuals, the critical state is a feature of network dynamics that is individual-invariant. Thus, it would be informative to study if the deviation from criticality can be used as an individual-invariant biomarker of network anomalies underlying WM deficits.

Our results quantitatively show that the excitatory currents are highly correlated with the inhibitory currents in individual neurons, indicating detailed E-I balance. The finding is consistent with the current-clamp recordings in single neurons that the excitatory and inhibitory currents of nearby cells track each other closely (Okun and Lampl, 2008). The current-clamp recording can only record the sum of the excitatory or inhibitory currents in a single neuron, so the relative contributions of sub-components of the currents, such as the recurrent currents, the external inputs, and the leaky currents of neurons, are difficult to delineate. Simulations are free from this limitation, which enables us to illustrate that the recurrent currents play a more important role in keeping the E/I balance compared to the background noise, the leaky currents, and item-targeted external inputs.

The most important finding of the present work is that optimal states at different scales in the network model are different expressions of the same underlying condition modulated by dopamine. The measures at each scale are characterized by a series of U or inverted-U profiles, and each extremum indicates an optimal working state in the corresponding scale. Specifically, at the system level, DA-mediated WM performance profiles suggest an optimal state accompanied by the maximum sensitivity and flexibility. At the network level, the optimal state is corresponding to the critical dynamics, which hold numerous advantages in information processing, including transmission (Beggs and Plenz, 2003; Rämö et al., 2007), storage (Haldeman and Beggs, 2005; Larremore et al., 2011), computation (Bertschinger and Natschläger, 2004), stability (Beggs, 2008) and dynamic range (Hosaka et al., 2008; Shew et al., 2009; Gautam et al., 2015). At the cellular level, an optimal state means that the excitatory and inhibitory currents most closely track each other (Okun and Lampl, 2008; Cafaro and Rieke, 2010), which can optimize the coding and metabolic efficiency (Yizhar et al., 2011; Sengupta et al., 2013), and track the external input more quickly (van Vreeswijk and Sompolinsky, 1996). Importantly, all these optimal states in the PFC manifested at different scales are regulated by the same control parameter—the concentration of dopamine at the synaptic level, with an intermediate concentration corresponding to above mentioned optimal states at all levels. An interesting question is that what is the mechanism to dynamically modulate the dopamine in the PFC. Anatomical studies indicate that the PFC contains abundant DA receptors (Goldman-Rakic, 1995) and receives diffuse projections from the mesocortical and mesolimbic dopaminergic systems originating in the ventral tegmental area of the midbrain (Bannon and Roth, 1983; Cools and D'Esposito, 2011). Thus, DA release in the PFC will occur in response to a variety of events either aversive or rewarding, and this release may prepare the PFC networks running in the optimal state to deal with environmental or cognitive challenges (Seamans and Robbins, 2010).

Working memory deficits were observed in many brain disorders, such as schizophrenia (Lett et al., 2014), depression (Austin et al., 2001), epilepsy (Swartz et al., 1996), and autism (Steele et al., 2007). At the same time, abnormal regulation in dopamine has been reported in related disorders, especially in schizophrenic patients (Abi-Dargham et al., 2002). In addition, disruption in the E/I balance has been implicated in the same set of diseases (Rubenstein and Merzenich, 2003; Fritschy, 2008; Marín, 2012; Murray et al., 2014). Here we provide a cross-scale view to better understand how the changes in dopamine in the PFC might cause E/I imbalance, which can push the network away from the critical state and eventually induce WM impairments. This provides a potentially useful multi-scale framework to reveal how the effects of abnormal neuromodulation at the synaptic level can penetrate different scales and give rise to functional deficits in different pathological conditions.

Our results indicate that these optimal states in the PFC manifested at different scales are actually multi-scale expressions of the same condition modulated by dopamine. More generally, the multi-scale nature of complex biological systems are widely reported. For example, healthy heartbeat interval series have been found to exhibit multi-fractal properties (Ivanov et al., 1999, 2004). In the brain, activity measures across a wide range of spatial scales, including those based on neural spikes, the local field potential (LFP), magnetoencephalography (MEG), functional magnetic resonance imaging (fMRI), have revealed a highly similar dynamical regime close to criticality (Beggs and Plenz, 2003; Tagliazucchi et al., 2012; Shriki et al., 2013; Bellay et al., 2015). In addition, previous modeling study have also reported pervasive scaling laws at the cellular, network and behavioral levels in the critical branching neural network (Kello, 2013). The current results further highlight that incorporating the multi-scale properties with a cross-scale perspective is vital for understanding complex phenomena and processes in physiology.

In summary, based on studying a neural network model, here we demonstrate a cross-scale mechanism of dopamine modulation for state optimization in the PFC, which for the first time links several seemingly unrelated regularities at different levels into a unified, coherent framework. Our results suggest that the optimal performance of WM at the system level, critical dynamics at the network level, and E/I balance at the cellular level could be multi-scale expressions of one optimal state in the PFC. This unified framework gives a novel cross-scale understanding of state optimization in the PFC, and more generally, provide a new perspective to incorporate scale-specific regularities into a coherent, cross-scale account for brain functions.

## Materials and Methods

### Network Model

Our model was adapted from a biophysically realistic model proposed by previous work (Mongillo et al., 2008), which utilizes calcium-mediated synaptic facilitation among recurrently connected excitatory neurons to form WM.

#### Model Architecture

The architecture of our model is shown in Figure 1A. The network is composed of three types of neuronal populations: (1) selective excitatory (Es) populations (from Es1 to Esp) to encode in total *p* memory items, each containing *fN*_*E*_ neurons selected from a pool of excitatory neurons (in total *N*_*E*_ = 8,000 excitatory neurons; *f* is a proportion common to all Es populations); (2) one non-selective excitatory (Ens) population formed by the remaining (1 – *pf*)*N*_*E*_ excitatory neurons; and (3) one inhibitory population (I) with *N*_*I*_ = 2,000 inhibitory neurons.

Each neuron, regardless of which population it belongs to, randomly receives presynaptic connections from all other neurons in the network with common probability *c*. To mimic the long-term potentiation effect of Hebbian learning, the excitatory-to-excitatory connections (*J*_*EE*_) within the same Es population are set to be stronger (*J*_*p*_), whereas the connections between different Es populations are set to be weaker, i.e., baseline value (*J*_*b*_). The synapses connecting neurons from the Es populations to neurons in the Ens population, as well as the connections within the Ens population, take the potentiated strength (*Jp*) with probability γ and the baseline strength (*J*_*b*_) with probability (1 – γ). These synaptic strengths are indicated by *J*_*b*/*p*_ in Figure 1A.

All excitatory-to-excitatory synapses (*J*_*EE*_) display short-term plasticity (see below), whereas the remaining synapses, including the excitatory-to-inhibitory (*J*_*EI*_), inhibitory-to-excitatory (*J*_*IE*_), and inhibitory-to-inhibitory (*J*_*II*_), are constant. For convenience, all parameters used in this model are listed in Table 1.

**Table 1:** Parameters used in the model.

**Dopamine modulation parameters**		**Dopamine1**	**Dopamine2**
Optimal concentration of dopamine	*D_0_*	1.0	1.0
Concentration domain	[*Dmin, Dmax*]	[0, 2.0]	[0, 2.0]
Steep parameter	*K_*c*_*	0.150	0.120
Shifting parameter	*D_*v*_*	0.105	0.185
**Selective stimulation**		**Strength1**	**Strength2**
Contrast factor	*A_*cue*_*	1.10	1.15
**Network parameters**		**E**	**I**
Coding probability	*f*	0.10	0.10
Number of memory items	*p*	5	5
Probability of synaptic connection	*c*	0.20	0.20
Number of excitatory/inhibitory cells	*N*	8,000	2,000
Mean external current	μ_*ext*_	23.80 mV	21.0 mV
Standard deviation of external current	σ_*ext*_	1.0 mV	1.0 mV
**Cell parameters**		**E**	**I**
Spike emission threshold	θ	20 mV	20 mV
Reset potential	*V*_*r*_	16 mV	13 mV
Membrane time constant	τ	15 ms	10 ms
Absolute refractory period	τ_*arp*_	2 ms	2 ms
**Synaptic parameters**		**Values**	
Synaptic efficacy E to I	*J_*EI*_*	0.135 mV	
Synaptic efficacy I to E	*J_*IE*_*	0.25 mV	
Synaptic efficacy I to I	*J_*II*_*	0.20 mV	
Baseline level of E to E synapses	*J_*b*_*	0.10 mV	
Potentiated level of E to E synapses	*J_*p*_*	0.45 mV	
Fraction of initial potentiated synapses	γ	0.10	
Synaptic delays	δ	0–1 ms	
**Short-term synaptic parameters**		**Values**	
Baseline utilization factor	*U*	0.20	
Baseline available resources	*X*	1.00	
Recovery time of utilization factor	τ_*d*_	1,500 ms	
Recovery time of synaptic resources	τ_*f*_	200 ms	

*Dopamine1 and Dopamine2 are parameters used for the white and gray modulation curve in Figure 1F, respectively. Some parameters are based on a previous computational working memory model (Mongillo et al., 2008)*.

#### Dynamic Rules of the Model

Activities of individual neurons are modeled by the leaky integrate-and-fire model (LIF) with a refractory period of τ_*arp*_. Below the firing threshold (θ), the membrane potential of neuron *i* (*V*_*i*_) is governed by:

(1) $$\begin{array}{r} \tau_{\text{m}}{\overset{\cdot }{V}}_{i}=-V_{i}+I_{i}^{\left(rec\right)}\left(t\right)+I_{i}^{\left(ext\right)}\left(t\right) \end{array}$$

where τ_m_ is the membrane time constant. The external input (including background noise and memory-specific stimuli) $I_{i}^{\left(ext\right)}\left(t\right)$ is modeled as Gaussian white noise, with a mean of μ_*ext*_ and standard deviation of σ_*ext*_:

(2) $$\begin{array}{r} I_{i}^{\left(ext\right)}\left(t\right)=\mu_{ext}+\sigma_{ext}\cdot \eta_{i}^{}\left(t\right) \end{array}$$

where $\eta_{i}^{}\left(t\right)$ is the standard Gaussian white noise. Memory-specific stimuli are modeled by increasing μ_*ext*_ but maintaining σ_*ext*_. The recurrent current $I_{i}^{\left(rec\right)}\left(t\right)$ is given by:

(3) $$\begin{array}{r} I_{i}^{\left(rec\right)}\left(t\right)=\underset{j}{\sum }{Ĵ}_{ij}\left(t\right)\underset{k}{\sum }\delta \left(t-t_{\text{k}}^{\left(j\right)}-D_{ij}\right) \end{array}$$

where $t_{\text{k}}^{\left(j\right)}$ refers to the firing times of presynaptic neuron *j*, *D*_*ij*_ is the transmission delay uniformly distributed between 0 and 1 ms, and Ĵ_*ij*_(*t*) is the instantaneous synaptic efficacy. For excitatory-to-excitatory synapses, their strengths are dynamically adjusted according to:

(4) $$\begin{array}{rcl} {Ĵ}_{ij}\left(t\right) & = & J_{ij}\cdot u_{j}\left(t-D_{ij}\right)x_{j}\left(t-D_{ij}\right) \end{array}$$

(5) $$\begin{array}{rcl} \overset{\cdot }{u}\left(t\right) & = & \frac{U-u_{j}\left(t\right)}{\tau_{f}}+U\left[1-u_{j}\left(t\right)\right]\underset{k}{\sum }\delta \left(t-t_{k}^{\left(j\right)}\right) \end{array}$$

(6) $$\begin{array}{rcl} {ẋ}_{j}\left(t\right) & = & \frac{X-x_{j}\left(t\right)}{\tau_{d}}+u_{j}\left(t\right)x_{j}\left(t\right)\overset{}{\underset{k}{\sum }}\delta \left(t-t_{k}^{\left(j\right)}\right) \end{array}$$

where *x* indicates the available number of presynaptic neurotransmitters, and *u* refers to the portion of *x* that can be utilized during synaptic transmission, which reflects the influence of calcium level on release probability at the presynaptic site. *U* and *X* are the baseline values for *u* and *x*, respectively. After each spike, *x* and *u* change according to Equations (5, 6) with their corresponding time constants τ_*d*_ (depressing) and τ_*f*_ (facilitating), respectively. As mentioned above, only the excitatory-to-excitatory synapses are subjected to this form of plasticity. All remaining synapse efficacies are kept constant.

#### Model Dopamine Modulation

Previous studies have shown that different levels of dopamine D1 receptor activation act differently on glutamatergic input between the excitatory and inhibitory neurons. Specifically, with low doses of DA, the inputs to both excitatory and inhibitory neurons are unaffected; with moderate doses of DA, the enhancement of glutamatergic input to excitatory neurons is more pronounced; and with high doses of DA, the inputs to both excitatory and inhibitory neurons are strongly enhanced(Muly et al., 1998; Gao et al., 2001). These differential effects of D1 activation level have been widely acknowledged in computation models studying DA modulation of the prefrontal cortical networks (Durstewitz et al., 2000; Brunel and Wang, 2001; Lew and Tseng, 2014). To reflect D1 modulation in the present model, we multiplied the absolute strength of excitatory-excitatory (E-E) synapses (*J*_*EE*_, including *J*_*b*_*, J*_*p*_, and *J*_*b*/*p*_) and excitatory-inhibitory (E/I) synapses (*J*_*EI*_) with relative strength factors *A*_*EE*_ and A_*EI*_, respectively. *A*_*EE*_ and A_*EI*_ are both functions of DA level, and the differences between them change with DA, as indicated by the red dotted line (*A*) in Figure 1E. The range of DA was set as *D* ∈ [0, 2]. Accordingly, we fixed the intermediate level of D1 activation at the center of the range, i.e., *D*_0_ = 1.0. The range of the two scaling factors was set as [*A*_*min*_, *A*_*max*_]. The functions of *A*_*EE*_ and *A*_*EI*_ could then be specified as:

(7) $$\begin{array}{rcl} A_{EE} & = & \left[\frac{1}{C_{EE}}\cdot \left(1+\frac{D_{L}}{1+e^{\left(D_{E0}-D\right)/K_{c}}}\right)-A_{P1}\right]\cdot 100\% \end{array}$$

(8) $$\begin{array}{rcl} A_{EI} & = & \left[\frac{1}{C_{EI}}\cdot \left(1+\frac{D_{L}}{1+e^{\left(D_{I0}-D\right)/K_{c}}}\right)-A_{P1}\right]\cdot 100\% \end{array}$$

(9) $$\begin{array}{rcl} D_{E0} & = & D_{0}-D_{V} \end{array}$$

(10) $$\begin{array}{rcl} D_{I0} & = & D_{0}+D_{V} \end{array}$$

(11) $$\begin{array}{rcl} D_{L} & = & A_{max}-A_{min} \end{array}$$

(12) $$\begin{array}{rcl} A_{P1} & = & 1-A_{min} \end{array}$$

where *D* refers to the level of D1 activation, and *K*_*C*_ and *D*_*V*_ are two key parameters controlling the function shape in Figure 1E. Steep parameter *K*_*C*_ controls the vertical steepness and shifting parameter *D*_*V*_ controls the largest efficacy difference between the two functions at *D*_*0*_. *C*_*EE*_ and *C*_*EI*_ are the normalization parameters obtained by setting *D*_*V*_ = *0* in Equations (9, 10) and *D* = *D*_*0*_ in the following equations:

(13) $$\begin{array}{r} C_{EE}=1+\frac{D_{L}}{1+e^{\left(D_{E0}-D\right)/K_{c}}} \end{array}$$

(14) $$\begin{array}{r} C_{EI}=1+\frac{D_{L}}{1+e^{\left(D_{I0}-D\right)/K_{c}}} \end{array}$$

Each D1 activation level uniquely determines a *A*_*EI*_*, A*_*EE*_ pair (Figure 1E), which then specifies a point in the EE-EI plane. With a continuously increasing D1 activation level, a system state trajectory can be obtained. The exact shape and position of the trajectory can be controlled by tuning parameters *K*_*C*_ and *D*_*V*_. Given (1) the objective function of obtaining the maximal sensitivity of WM and avoiding the risk of “imaginary memory” (see the main text for details), and (2) the constraints that at very low/high D1 activation both *J*_*EE*_ and *J*_*EI*_ should be weak/strong, eventually, we can obtain the trajectory as shown in Figure 1F. With the increase in D1 activation level, the trajectory starts from the diagonal, then approaches the transition line, before finally returning to the diagonal.

Our main conclusions are robust toward different choices of free parameters used in the model. For example, we show that similar results (Supplementary Figure S1) can be obtained with a different range of synaptic strengths, controlled by the scaling factor [*A*_min_, *A*_max_], i.e., the gray trajectory in Figure 1F.

### Analysis of Network Activities

#### Neuronal Avalanche Identification

We defined neuronal avalanches according to previous work (Beggs and Plenz, 2003) (cf. Figure 3A). An avalanche is a cascade of activity propagation within the network. To identify such a cascade, a small time-window Δ*t* is used to bin a Es population activities. An inactive time bin is a bin during which no neurons have fired, whereas an active time bin is the one during which at least one neuron has fired. The cascades are then defined as neuronal activities that occur either within the same bin or within consecutive active bins (Figure 3A). For the present analysis, Δ*t* = 0.3 ms, which is two times the average inter-spike interval within the population. Avalanche size is defined as the number of neurons involved in the corresponding cascade. All neuronal avalanches in the present work are obtained with 2,000 ms-long simulations with a time step of 0.1 ms.

#### Power-Law Fitting

In critical neural networks, avalanche size distribution follows a power law:

(15) $$\begin{array}{r} P\left(s\right)\propto s^{\alpha } \end{array}$$

where P(*s*) is the probability density function (PDF) of observed avalanche size *s*, α is the exponent that gives the power-law slope in a log-log plot, which is close to −1.5 for critical networks measured under both *in vitro* and *in vivo* conditions (Beggs, 2004; Gireesh and Plenz, 2008). To reduce the effect of noise on distribution, a smoothing method based on geometric mean values under the log-log coordinate was applied (Christensen and Moloney, 2005), and the fit area are obtained by optimization the object of minimal the Kolmogorov–Smirnov distance between the data and fitting candidates, finally the exponent α then estimated by least-square fitting in log-log coordinates. We implemented these through a public python package Powerlaw (Alstott et al., 2014).

#### Branching Parameter

Branching parameter σ is defined as the average number of active units in the next time step that are triggered by one active unit at the current time step. Following previous work (Beggs and Plenz, 2003), it can be measured by:

(16) $$\begin{array}{r} \sigma \text{=}\langle \frac{Descendants}{Ancestors}\rangle \end{array}$$

where 〈〉 refers to the operation of arithmetic average, *Ancestors* is the number of active units in the first bin of an avalanche, and *Descendants* is the number of active units in the second bin of the corresponding avalanche.

### Correlation Coefficient and Coefficient of Variation

The correlation coefficient is used to measure the degree of balanced excitatory and inhibitory input to a neuron, which is calculated as:

(17) $$\begin{array}{r} \rho =\frac{\mathrm{cov}\left(x_{1},x_{2}\right)}{\sqrt{D\left(x_{1}\right)+D\left(x_{2}\right)}} \end{array}$$

where *x*_*1*_ and *x*_*2*_ refer to the excitatory and inhibitory currents, respectively, and *D* is the variance of the corresponding currents. Note that the correlation coefficient between excitatory and inhibitory currents is always a negative value. We always plotted its absolute value in this to facilitate visual comparison.

The coefficient of variation of distribution is defined as:

(18) $$\begin{array}{r} CV=\frac{\mu }{\sigma } \end{array}$$

where μ and σ refer to the mean and standard deviation of distribution, respectively.

## Author Contributions

GH, XH, TJ, and SY planned the study. GH and XH carried out network simulations and analysis. GH and SY wrote the manuscript.

### Conflict of Interest Statement

The authors declare that the research was conducted in the absence of any commercial or financial relationships that could be construed as a potential conflict of interest.
