# Supplementary material for: Multi-Scale Expressions of One Optimal State Regulated by Dopamine in the Prefrontal Cortex
Source: Front Physiol. 2019 Feb 28;10:113. doi: 10.3389/fphys.2019.00113 (PMC6404637; doi:10.3389/fphys.2019.00113)
Supplement: Supplementary file 1 [file Data_Sheet_1.PDF]

Supplementary Material

Multi-scale Expressions of One Optimal State Regulated by Dopamine in the Prefrontal Cortex

Guyue Hu, Xuhui Huang, Tianzi Jiang, Shan Yu*

*** Correspondence:** Shan Yu (shan.yu@nlpr.ia.ac.cn)

1 Supplementary Figure

Figure S1. Results obtained with a different strength range $[A_{min}, A_{max}]$. Behaviors at different levels corresponding to the grey trajectory in Fig. 1F are shown. All analyses and results are parallel to the ones in the main text. For working memory at the system level, A-B are similar to Fig. 2B-C, and C-G are similar to Fig. 2E-I. For criticality at the network level, H-I are similar to Fig. 3C-D. For detailed balance of excitatory and inhibitory at the cellar level, J-M are similar to Fig. 4A-D.

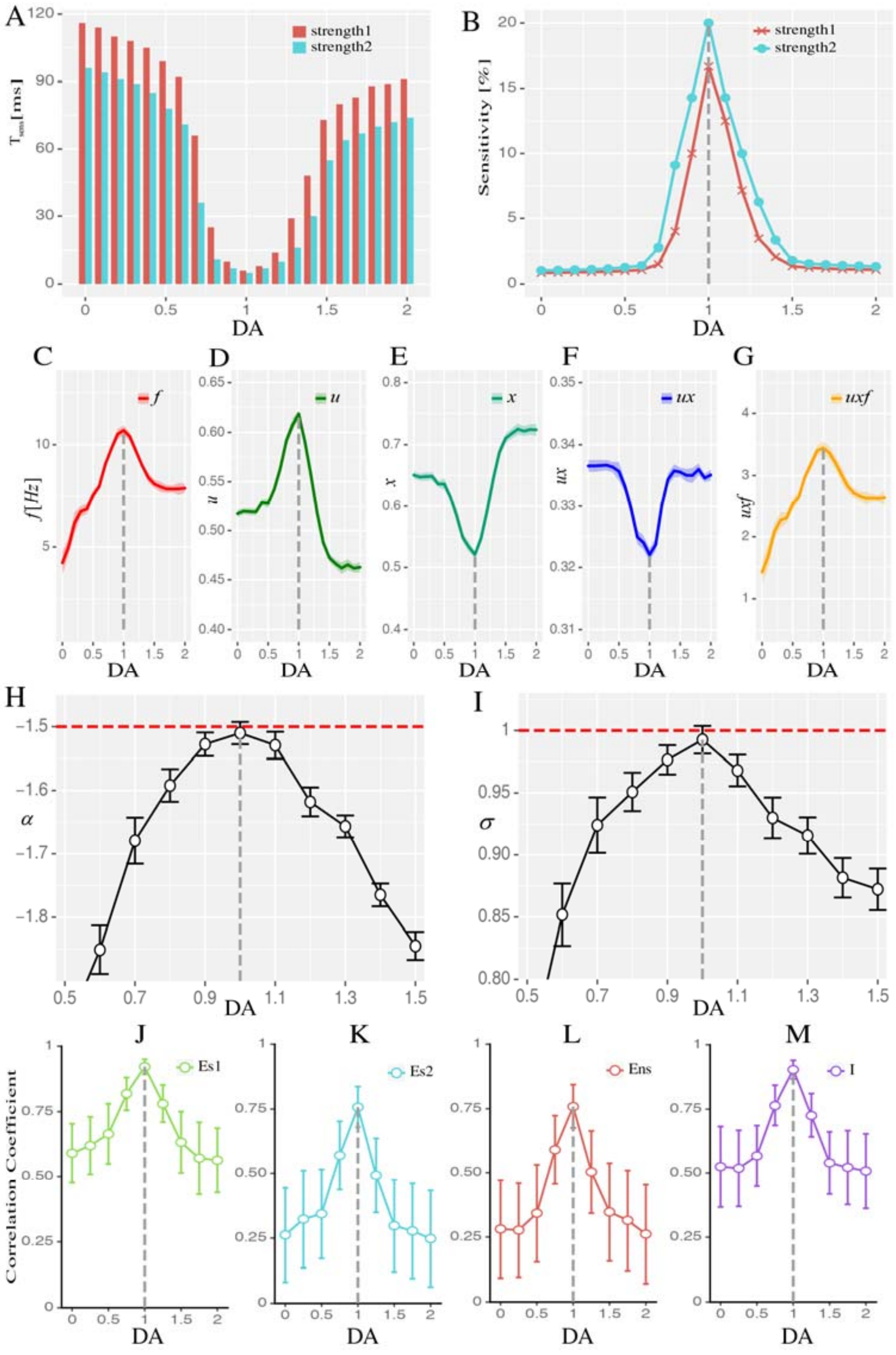

A
B
C
D
E
F
G
H
I
J
K
L
M
strength1
strength2
T_{sim}[ms]
Sensitivity [%]
DA
f
u
x
ux
uxf
f [Hz]
α
σ
Correlation Coefficient
Es1
Es2
Ens
I

Fig. S1
